# Antioxidant and Cell Proliferation Properties of the Vietnamese Traditional Medicinal Plant *Peltophorum pterocarpum*

**DOI:** 10.3390/molecules25204800

**Published:** 2020-10-19

**Authors:** Seon-Rye Kim, Dao Cuong To, Phi Hung Nguyen, Yen Nhi Nguyen, Byung-Jun Cho, Manh Hung Tran

**Affiliations:** 1 Department of Pharmacy, College of Pharmacy, Kangwon National University, 1 Gangwondaegakgil Chuncheon-si, Gangwon-do 24341, Korea; sjsanj@hanmail.net; 2 Faculty of Pharmacy, Phenikaa University, Yen Nghia, Ha Dong district, Hanoi 12116, Vietnam; cuong.todao@phenikaa-uni.edu.vn; 3 Phenikaa Research and Technology Institute (PRATI), A&A Green Phoenix Group JSC, 167 Hoang Ngan, Cau Giay District, Hanoi 11313, Vietnam; 4 Institute of Natural Products Chemistry, Vietnam Academy of Science and Technology (VAST), 18 Hoang Quoc Viet street, Cau Giay District, Hanoi 122100, Vietnam; nguyenphihung1002@gmail.com; 5 University of Science, Vietnam National University Hochiminh City, 227 Nguyen Van Cu street, District 5, Ho Chi Minh City 748000, Vietnam; yennhinguyen2507@gmail.com; 6 Department of Emergency Medical Technology, College of Health Science, Kangwon National University, 346 Hwangjo-gil, Dogye-up, Samcheok-si, Gangwon-do 25945, Korea

**Keywords:** *Peltophorum pterocarpum*, anti-oxidant, LDL, human leukemia cancer cells, caspases

## Abstract

*Peltophorum pterocarpum* is regarded as one of the most important medicinal plants in the traditional medicine system of Vietnam. However, scientific evidence for the antioxidant effects against lipid peroxidation and the potential effects in cancer of this plant are lacking. In our experiments, 70% ethanolic extracts of *P. pterocarpum* leaves (LPP) and stem bark (SPP) were evaluated for their low-density lipoprotein (LDL) oxidation and cytotoxic activity against cancer cell lines. Both LPP and SPP inhibited Cu^2+^-mediated LDL by increasing the lag time of conjugated diene formation and inhibiting the generation of thiobarbituric acid reactive substances (TBARS) in a dose-dependent manner. In cancer cells, LPP and SPP triggered the most potent cytotoxic effects against human leukemia cells, CRF-SBA and HL-60, with half-maximal inhibitory concentration (IC_50_) values ranging from 118.5 to 157.2 µg/mL. SPP exhibited significant cytotoxicity against MIA PACA2, A549, and KG cell lines with IC_50_ values of 167.5, 244.1 and 255.0 µg/mL, respectively. Meanwhile, LPP showed cytotoxic activity against KG with an IC_50_ value of 228.1 µg/mL. SPP mediated cytotoxicity in HL-60 and CCRF-SBA cells through the activation of the apoptosis pathway, including the activation of caspases 3, and 9 and poly (ADP-ribose) polymerase (PARP). These results suggested that SPP may prevent the development and progression of atherosclerosis and leukemia in humans.

## 1. Introduction

*Peltophorum pterocarpum* (Lim Xẹt or Muồng Kim Phượng Vàng in Vietnamese) belongs to the Fabaceae family and is native to tropical, southeastern Asian countries such as Thailand, Laos, Vietnam, India, and Sri Lanka, and Australia [1,2]. This plant is a popular ornamental tree, grown around the world. The leaves of *P. pterocarpum* are bipinnate, the flowers are yellow, and the fruit is initially red, then ripens to black, containing one to four seeds [2]. Traditionally, the bark of this tree was used for the treatment of dysentery, as an eye lotion, and as an embrocation for pains and sores. The leaf decoction was used to treat skin disorders; the stem bark was used to treat dysentery, for gargling applications, as a tooth powder, and to treat muscular pain. Its flowers were also used to cure or relieve intestinal disorders [3]. In Chinese traditional medicine, this plant has been used for the treatment of several ailments, including stomatitis, insomnia, skin troubles, constipation, ringworm, insomnia, dysentery, muscular pains, sores, and skin disorders [4]. *P. pterocarpum* is known to be a rich source of aliphatic alcohols, fatty acids, amino acids, terpenoids, phenolics, flavonoids, alkaloids, and steroids [4,5]. The isolated phytochemicals and various extracts from this plant have exhibited numerous biological activities, including antimicrobial, antioxidant, and cytotoxic activities, aldose reductase inhibition, and antiglycemic activities [6,7,8,9,10]. A 70% ethanolic extract of *P. pterocarpum* leaves displayed hepatoprotective effects in paracetamol-induced acute liver damage in albino Wister rats. This ethanolic extract (100 mg/kg and 200 mg/kg) significantly decreased tissue lipid peroxidation in rats. These results suggest that the *P. pterocarpum* extract might have value for the treatment of paracetamol-induced hepatic damage and some liver diseases, via its antioxidant effects [11]. *P. pterocarpum* and *Morinda lucida,* combined, were evaluated for their cognitive enhancing potential in scopolamine-induced amnesic animals; the two ingredients resulted in the considerable enhancement of cognition in mice [12]. The aqueous extract of *P. pterocarpum* wood also exhibited inhibitory effects against Epstein-Barr virus (EBV) in Raji cells and melanogenesis in α-melanocyte-stimulating hormone (α-MSH)-stimulated B16 melanoma cells, and demonstrated potent free radical-scavenging activity. Additionally, bergenin and gallic acid, isolated from *P. pterocarpum*, exhibited potent inhibitory effects against EBV-early antigen activation and skin tumor promotion in an in vivo, two-stage, mouse skin carcinogenesis test, using 7,12-dimethylbenz[a]anthracene as an initiator and tissue plasminogen activator (TPA) as a promoter [13].

In our search for antioxidant and anticancer agents derived from Vietnamese natural and medicinal plants, we have found that extracts of the leaves (LPP) and stem bark (SPP) of *P. pterocarpum* show significant activities [14]. This study attempts to investigate the LDL oxidative and cytotoxic effects of both LPP and SPP in some human cancer cell lines.

## 2. Results

**Figure 1:**
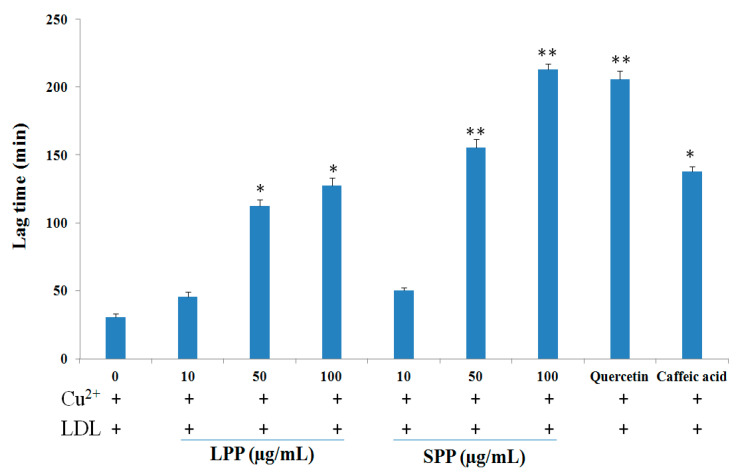
Effects of LPP and SPP on kinetic of Cu^2+^ mediated LDL oxidation. Data were expressed as mean S.D. values for *n* = 3. * *p* < 0.01, ** p < 0.05 compared to negative control. Quercetin (5 µM) and caffeic acid (5 µM) were used as positive controls.

The oxidative modification of LDL is thought to be an essential factor for the initiation and development of atherosclerosis. Because the formation of conjugated dienes represents the initial phase of LDL oxidation, lag time represents an indicator of the oxidation-resistant capacity of LDL [15]. In our experiment, the effects of LPP and SPP on the lengthening of the lag time were measured, as shown in Figure 1. When the LDL was incubated with Cu^2+^ alone, the lag time was recorded at 30.3 ± 2.5 min. However, treatment with LPP extract (10, 50, and 100 µg/mL) significantly increased the lag period for LDL oxidation to 45.5 ± 3.8, 112.2 ± 5.7, and 127.6 ± 4.5 min, respectively. Interestingly, different SPP extracts, at doses of 10, 50, and 100 µg/mL, inhibited the oxidation in LDL by retarding the lag time to 50.2 ± 2.2, 155.6 ± 5.8, and 212.7 ± 4.6 min, respectively. In this experiment, quercetin (5.0 µM) and caffeic acid (5.0 µM), which were used as positive controls, extended the lag time to 205.5 ± 6.5 and 137.0 ± 3.8 min, respectively (Figure 1).

To quantify LDL oxidation, oxidation was initiated by incubation with Cu^2+^, and the formation of malondialdehyde (MDA) was assessed using the TBARS assay [16]. Both LPP and SPP reduced the formation of TBARS in dose-dependent manners, with half-maximal inhibitory concentration (IC_50_) values of 67.5 ± 3.6 and 48.0 ± 5.2 µg/mL, respectively. Quercetin, caffeic acid, and α-tocopherol were used as reference antioxidants, which showed inhibitory effects against Cu^2+^-mediated LDL oxidation, with IC_50_ values of 3.5 ± 0.8, 5.7 ± 1.4, and 25.8 ± 2.5 µM, respectively (Table 1). A variety of other factors also promoted the lipid oxidation of LDL. In this study, we used another mediator, 2,2′-azobis(2-amidinopropane) dihydrochloride (AAPH), a hydrophilic peroxyl radical, as a second activation source. AAPH is a thermo-labile radical initiator and water-soluble azo agent that is used extensively as a free radical generator, particularly in the study of lipid peroxidation and the characterization of antioxidants. The formation of MDA by AAPH (Table 1) was reduced by LPP and SPP treatment in the TBARS assay, with IC_50_ values of 112.7 ± 4.5 and 40.5 ± 3.2 µg/mL, respectively. Quercetin, caffeic acid, and α-tocopherol showed inhibitory effects against AAPH-mediated LDL oxidation, with IC_50_ values of 10.5 ± 1.0, 40.7 ± 1.8, and 20.4 ± 1.0 µM, respectively.

Two human pancreatic cell lines, MIA PACA2 and PANC-1, a human lung cancer cell line, A549, an ovarian cancer cell line, OVCAR8, and three leukemia cancer cell lines, KG, HL-60, and CCRF-SBA, were used in this study. To determine the cytotoxic effects of LPP and SPP in cancer cell lines, a cell proliferation assay was performed using a cell counting kit. The results in Table 2 show that SPP exhibited cytotoxicity against the MIA PACA2, with an IC_50_ of 167.5 ± 5.6 µg/mL; in contrast, LPP showed very weak cytotoxicity, with an IC_50_ greater than 500 µg/mL. SPP also inhibited the growth of A549 cells, with an IC_50_ of 244.0 ± 11.5 µg/mL. LPP and SPP showed very weak cytotoxic activity against PANC-1 and OVCAR8 cells. However, both LPP and SPP showed potent cytotoxicity against human leukemia cancer cells, such as KG, HL-60, and CCRF-SBA cells, demonstrating IC_50_ values ranging below 255.0 ± 8.1 µg/mL (Table 2). SPP showed the most potent cytotoxicity against HL-60 cells, with an IC_50_ of 118.5 ± 3.8 µg/mL. This result supported the reported traditional use of *P. pterocarpum* for the treatment of leukemia. Additionally, neither LPP nor SPP demonstrated cytotoxicity in normal human embryonic kidney cells (HEK293, data not show).

**Figure 2:**
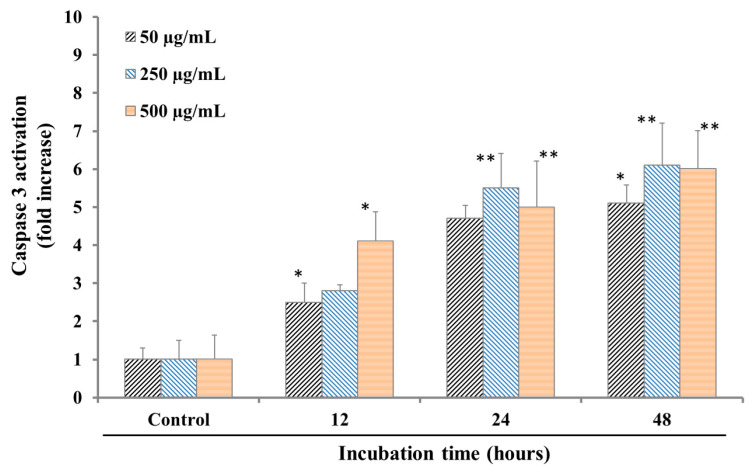
Effect of SPP on caspase-3 activation in HL-60 cells. HL-60 cells (1 × 10^6^/well) were incubated with SPP (50–500 μg/mL). The cell lysates were incubated at 37 °C with caspase-3 substrate (Ac-DEVD-AFC) for 1 h. The fluorescence intensity of the cell lysates was measured to determine the caspase-3 activity. The blank group was used as 0.1% DMSO-treated cells. Data are presented as the mean ± SD of results from three independent experiments (* *p* < 0.01; ** *p* < 0.05).

SPP was, consequently, selected for additional enzymatic experiments in HL-60 leukemia cancer cells, due to its cytotoxicity potential. SPP (50–500 µg/mL) was added to HL-60 cells, followed by incubation for 12, 24, and 48 h. Then, caspase 3 activation was measured according to the levels of Ac-Asp-Glu-Val-Asp-8-amino-4-trifluoromethylcoumarin (Av-DEVD-AFC). The results in Figure 2 show that SPP (50 µg/mL) activated caspase 3, increasing the cleaved enzyme level by 2.5- and 4.5-fold after 12 and 24 h, respectively, and the cleaved, activated form of caspase 3 by five-fold compared to the control (*p* < 0.01, Figure 2). SPP treatment (250 µg/mL) increased the level of activated caspase 3 between 3- and 6-fold after 12, 24, and 48 h of incubation compared with the levels in controls (*p* < 0.05). SPP treatment (500 µg/mL) increased activated caspase 3 levels between 4- and 8-fold after 48 h of incubation compared with the levels observed in the control (*p* < 0.05). These results suggested that SPP could increase the activated form of caspase 3, in a dose- and time-dependent manner.

**Figure 3:**
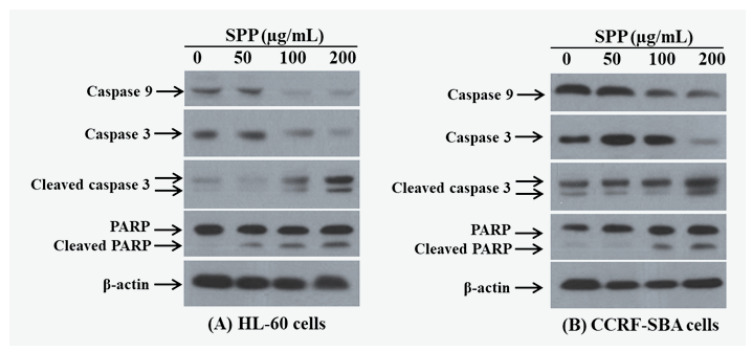
Influences of SPP on regulation of apoptotic signal proteins in HL-60 (**A**) and CCRF-SBA (**B**) cells. All experiments were repeated at least three times, obtaining similar results. Both cells were treated with SPP (0–500 µg/mL) for 48 h. β-Actin was used as a control, (–), 0.1% DMSO-treated cells. The experiments were carried out in three replicates.

To determine the cell apoptosis signaling pathway that is triggered by SPP, we investigated alterations in mitochondria-associated signal proteins, such as caspases 3, 9, and poly (ADP-ribose) polymerase (PARP). Caspase 3 is a known member of the cysteine-aspartic acid protease family, which exists as an inactive, pre-enzymatic precursor form of 32 kDa in size. When activated, caspase 3 cleaves multiple structural and regulatory proteins, leading to death by triggering the apoptosis pathway via the cleavage of proteins into heterozygous substances [17]. While caspase 3 is considered the executioner of cells, caspase 9, the other cysteine-aspartic acid protease found in humans, and which is encoded by *CASP9*, is an initiator of cell death [17,18]. In our experiment, SPP was added to HL-60 and CCRF-SBA cells and incubated for 48 h, after which the levels of the apoptotic proteins caspase 3 and caspase 9 were detected in HL-60 and KG cells by western blotting analysis. SPP (0–500 µg/mL) cleaved and activated caspase 3 and caspase 9 into their cleaved forms. The results showed that SPP significantly inhibited the expression of these caspase enzyme proteins in a dose-dependent manner (Figure 3). To further evaluate whether SPP could induce apoptosis in HL-60 and CCRF-SBA cells, the levels of cleaved PARP were measured after treatment with SPP (0–500 µg/mL) using western blot analysis. PARP represents a protein family that plays a vital role in many cellular processes, such as DNA repair, genomic stability, and programmed cell death [19]. Catalyzing caspase-3 activation generally leads to the cleavage of PARP, a process that plays a positive regulatory role in the apoptosis of several cells. It is considered to be a biomarker for the detection of apoptosis [20]. As shown in Figure 3, SPP (0–500 µg/mL) induced HL-60 and CCRF-SBA cell apoptosis in a dose-dependent manner, associated with PARP cleavage.

**Figure 4:**
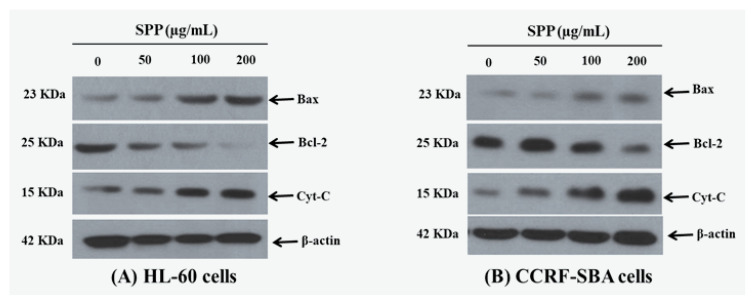
Effects of SPP on regulation of Bcl-2 family proteins of HL-60 (**A**) and CCRF-SBA cells (**B**). Data are representative of three independent experiments.

We further investigated the effects of SPP (0–500 µg/mL) on the levels of the pro-apoptotic protein Bax, the anti-apoptotic protein Bcl-2, and the mitochondrial-damage associated protein cytochrome c (Cyt-c) [21]. Cyt-c is discharged from the mitochondria into the cytoplasm, triggering the apoptotic progression in HL-60 and CCRF-SBA cancer cells. The pro-apoptotic Bax and the anti-apoptotic Bcl-2 proteins belong to the Bcl-2 protein family, which controls the mitochondrial apoptotic pathway [22]. Here, we observed that SPP (0–500 µg/mL) treatment increased Bax protein expression levels and decreased Bcl-2 protein expression levels in both HL-60 and CCRF-SBA cancer cells in a dose-dependent manner. The results were also confirmed by western blot analysis, which demonstrated that the protein expression level of Cyt-c in the mitochondria was dose-dependently decreased (Figure 4).

## 3. Discussion

In recent years, studies have been performed to examine various plant-derived natural extracts, which are safe to use as antioxidant, anticancer, and other disease treatments, with fewer side effects than pharmaceutical products. In our experiment, ethanolic extracts of both the leaves and stem bark of *P. pterocarpum* were found to be active in lipid peroxidation and cytotoxicity. Previously, *P. pterocarpum* was found to contain many substances, such as phenolic compounds, flavonoids, flavonoid glycosides, terpenoids, alkaloids, and steroids [4,5]. Leaf and bark extracts of *P. pterocarpum*, collected in Thailand, were tested for their 2,2-diphenyl-1-picrylhydrazyl (DPPH), ABTS, and galvinoxyl radical-scavenging activities, phenolic contents, α-glucosidase, α-amylase, and aldose reductase inhibition, and the inhibition of advanced glycation end-product formation. These extracts showed significant antiglycemic activity, and the main active compound in the leaf was determined to be quercetin-3-O-β-d-galactopyranoside [23]. Bergenin, a C-glycoside of 4-*O*-methyl gallic acid isolated from *P. pterocarpum*, showed antioxidant activity against DPPH and hydroxyl and nitric oxide scavenging activities. Bergenin was identified as a mild antioxidant, which could be used as a natural antioxidant [24]. Bergenin and gallic acid were also isolated from the ethyl acetate (AcOEt)-soluble fraction obtained from *P. pterocarpum* collected in Japan; they exhibited inhibitory effects against EBV-early antigen activation and against skin tumor promotion in in vivo, two-stage, mouse skin carcinogenesis tests. Bergenin also exhibited melanogenesis inhibitory activities in α-MSH-stimulated B16 melanoma cells [13]. *P. pterocarpum,* together with several Malaysian plants, showed potent antioxidant activities but were not cytotoxic activity against 3T3 and 4T1 cells, even at concentrations as high as 100 µg/mL [7]. The physiological effects of *P. pterocarpum* extract, such as antioxidant, antihepatitis virus, antibacterial, and anticancer activities, have been reported. However, studies on antioxidant efficacy using LDL and anticancer in human leukemia cells have not been widely published.

To the best of our knowledge, our study is the first report describing the inhibition of LDL oxidation by both the leaves and stem bark of *P. pterocarpum*. Oxidation represents an important biochemical process, and is involved in the production of cellular energy for all biological processes in the human body. In atherosclerosis, the LDL oxidation process represents a type of liquid lipid peroxidation due to an excess of free radical and other oxidant species derived from oxygen, nitrogen, and other chemical elements in the body. The oxidation of LDL is thought to promote atherosclerosis by accelerating inflammatory reduction and changing immunologic mechanisms, resulting in lipid dysregulation and foam cell formation. The oxidation of LDL results in the formation of a complex between LDL, beta 2 glycoprotein I, and C-reactive protein in the intima of atherosclerotic lesions. Therefore, the inhibition of LDL modification is thought to be an essential target for the prevention of atherosclerosis. Our results showed that LPP and SPP could retard the lag time associated with the lipid peroxidation of LDL induced by Cu^2+^. In addition, SPP strongly inhibited oxidation by reducing the conjugated diene in LDL, which may be associated with the higher phenolic contents in the stem bark than in the leaves of *P. pterocarpum*.

The effects of *P. pterocarpum* on cancer cells were examined. Two human pancreatic cell lines, MIA PACA2 and PANC-1, a human lung cancer cell line, A549, an ovarian cancer cell line, OVCAR8, and three leukemia cancer cell lines, KG, HL-60, and CCRF-SBA, were treated with a range of *P. pterocarpum* extract concentrations. Biocompatibility was evaluated using a cell counting Dojindo kit. As shown in Table 1, both the LPP and SPP demonstrated cytotoxicity against human leukemia cancer cells. However, SPP showed the most potent activity against HL-60 cells compared with the other cells and compared with LPP. SPP was selected to investigate the effects at the molecular protein level in leukemia HL-60 and CCRF-SBA cells. Caspases 3 and 9 represent members of the cysteine-aspartic protease enzyme family and play essential roles in apoptosis. Caspases 3 and 9 are generally associated with the arrest of cell growth and development, and are called the “executors” of both normal and cancer cells. Activated caspase 9 then activates caspase 3, which releases cytochrome-C (Cyt-C), a mitochondrial-damage associated cytochrome, resulting in cell death through apoptosis and necrosis pathways [25,26]. Caspases are also responsible for cleaving numerous cellular targets, including members of the PARP family. PARP proteins are involved in several cellular processes, such as DNA repair, genomic stability, and programmed cell death. Recently, the importance of natural and medicinal plants and their novel, small-molecule compounds in cancer chemotherapy has been well-recognized. Several natural extracts and phytochemical compounds can induce apoptotic cancer cell death and could be employed for anticancer therapies. SPP activated caspase 3 and caspase 9, resulting in an increase in the cleaved forms, in a dose-dependent manner. The cleaved form of PARP also increased in both HL-60 and CCRF-SBA cells. The western blotting results showed that SPP, in a dose range from 50 to 500 µg/mL, was able to remarkably increase the protein levels of the pro-apoptotic Bax protein and cytosolic Cyt-c expression, but significantly decreased the protein levels of the anti-apoptotic protein Bcl-2. Bax and Bcl-2 are members of the Bcl-2 family and act as core regulators of the intrinsic apoptosis pathway through the mitochondrial regulation pathway.

Since *P. pterocarpum* was found to contain many phenolic compounds, flavonoids, terpenoids, and alkaloids, these secondary metabolites may be largely responsible for the anticancer activity of this plant. The isolation of potent active components and leukemia cell-based assays to identify the detailed mechanisms will be further explored. In addition, in vivo experiments will be performed to confirm the functionalities of these extracts will contribute to the development of *P. pterocarpum* and its phytochemicals as potential therapeutic agents for the treatment of human leukemia cancer.

## 4. Materials and Methods 

### 4.1. Plants Material

The leaves and stem barks of *Peltophorum pterocarpum* was collected at Danang city, Vietnam, in August 2017. The scientific name was identified by Professor Pham Thanh Huyen, PhD, Vietnam National Institute of Medicinal Material. A voucher specimen (TMH-41-2017A, and B) were deposited at the Laboratory of Medicinal Chemistry, Vietnam National University Hochiminh city.

### 4.2. Extraction and Isolation

The freshly collected plant materials as leaves and stem barks were dried in a hot air oven at 50 °C and then powdered. To prepare the extracts, 1 g of each powdered sample was extracted using 70% ethanol solvent using ultrasonic for three times (100 mL each). The combination extract was filtered and concentrated by complete evaporation in vacuo. The dried extract was then stored at −20 °C. The entire study was conducted using a single batch of each plant extract to avoid batch-to-batch variation and maximize product consistency.

### 4.3. LDL Preparation 

Venous blood was collected individually from healthy, normolipidemic volunteers. The plasma was centrifuged at 43,800 rpm for 20 h at 4 °C in Beckman T8M ultracentrifuge (Indianapolis, IN, USA) and then chylomicron and very low-density lipoprotein floating to the top of tube was removed. Other supernatants were collected, and adjusted to *d* = 1.063 g/mL using NaBr and centrifuged at 43,800 rpm for 24 h at 4 °C. The top layer of LDL was purified by fast protein liquid chromatography on a Sephacryl S4000HR column (16 mm × 600 mm, Marlborough, MA, USA) using 10 mM Tris–HCl buffer (pH = 7.4) containing 150 mM NaCl as the eluting solvent in the presence of 1 mM EDTA. The LDL was dialyzed against 50 μM phosphate-buffered saline (PBS) containing 1 μM EDTA for 24 h. EDTA was removed by a Sephadex G-25 column equilibrated with PBS. The purity of LDL evaluated by agarose gel electrophoresis was >97%. The LDL protein was determined by the bicinchoninic acid method using bovine serum albumin as standard [16].

### 4.4. Measurement of Conjugated Diene Formation 

The oxidation of LDL was assessed by the formation of conjugated dienes, determined as the change in UV absorbance at 234 nm [15]. Briefly, LDL (100 μg/mL) in PBS (pH 7.4, final volume of 1 mL) was pre-incubated with ethanolic extract of *P. pterocarpum* stem barks (SPP: 10, 50, 100 mg/mL) for 30 min at 37 °C, and then 5 μM CuSO_4_ was added to initiate the oxidation process. The lag time (minute) was determined as the intercept of baseline and the tangent of the absorbance curve during the propagation phase by those of absorbance at 234 nm that continuously monitoring at 10 min intervals for 5 h at 37 °C using a spectrophotometer (Shimadzu UV-1240, Tokyo, Japan). Quercetin and caffeic acid was used as positive controls [16,27].

### 4.5. Measurement of TBARS

For malondialdehyde (MDA) detection, LDL (100 μg/mL) in PBS was pre-incubated with LPP and SPP (10, 50, 100 mg/mL) as described above, and then 5 μM CuSO_4_ was added to initiate the oxidation. The reaction mixture was incubated at 37 °C for 3 h and the reaction was terminated by adding 250 μL of 20% trichloroacetic acid (TCA) and 250 μL of 1% thiobarbituric acid (TBA). After boiling at 95 °C for 5 min, the mixture was centrifuged at 10,000 rpm for 10 min. The absorbance of supernatant was measured at 532 nm (Shimadzu UV-1240, Tokyo, Japan). Quercetin and caffeic acid were used as reference antioxidants [16].

### 4.6. Cell Cultures and Cell Proliferation Assay

Human cancer cell lines, i.e., leukemia CCRF-SBA and HL-60, and lung cancer A549, ovarian cancer VCAR8, and pancreatic cancer MIA PACA2, PANC-1 obtained from American Type Culture Collection (ATCC, Manassas, VA, USA), were maintained in RPMI and EMDM 1640 (GibcoBRL, NY, USA) containing L-glutamine with 10% FBS and 1% penicillin–streptomycin. Cells were cultured at 37 °C in a 5% CO_2_ incubator. Cell proliferation activity was measured using Dojindo kit assay according to manual guidance. Viable cells were seeded in the growth medium (95 μL) into 96-well microtiter plates (1 × 10^4^ cells/well) and incubated at 37 °C in a 5% CO_2_ incubator. The test samples were dissolved in DMSO and diluted with the growth medium with a range concentration of 5–50 μg/mL. The final DMSO concentration was adjusted to <0.1%. After standing for 4 h, 10 μL of the test sample was added to each well. The same volume of medium with <0.5% DMSO was added to the control wells. Each sample was prepared in triplicate. After 48 h of the test samples treatment, 10 μL solution of Dojindo kit was added to the each well. After 4 h in the incubator, the optical density was measured at 540 nm (Molecular Devices, Sunnyvale, CA, USA). The IC_50_ value was defined as the concentration of sample which reduced absorbance by 50% relative to the vehicle-treated control [25].

### 4.7. Caspase-3 Activity

Caspase-3 enzyme activity was measured by proteolytic cleavage of the fluorogenic substrate Ac-DEVD-AFC by counting on a fluorescence plate reader (Twinkle LB970 microplate fluorometer, Berthold Technologies, Baden Württemberg, Germany). Cells (1 × 10^5 ^ cell/well) were treated with indicated samples at concentrations ranging from 50 to 500 μg/mL. After incubation for 24 h, cells were harvested and washed with cold PBS. The pellets were lyzed using 15 μL of lysis buffer [10 mM Tris-HCL (pH 8.0), 10 mM EDTA, 0.5% Triton X-100] at room temperature for 10 min, and then placed on ice; 100 μL of assay buffer [100 mM Hepes (pH = 7.5), 10 mM dithiothreitol, 10% (*w/v*) sucrose, 0.1% (*v/v*) Chaps, 0.1% (*v/v*) BSA], and 10 μL of substrate solutin (200 μM substrate in assay buffer) were added. After incubation at 37 °C for 1 h, fluorescence was measured with an excitation at 370 nm and an emission at 505 nm [25].

### 4.8. Western Blotting Analysis

Cancer cells (5 × 10^5^ cells/mL) were treated with extracts (5–50 μg/mL) for 24 h at 37 °C. Cell lysates were prepared in 100 µL of lysis buffer (Sigma, St. Louis, MO, USA) containing a protease inhibitor cocktail (Roche, Mannheim, Germany). Insoluble material was removed by centrifugation at 14,000 rpm for 10 min. Then, the protein contents in the supernatant were measured using a Bio-Rad DC protein assay kit (Bio-Rad, Hercules, CA, USA). The protein extracts (50 µg/well) were separated by SDS-PAGE and then transferred onto PVDF membranes (Bio-Rad, Hercules, CA, USA). The membranes were blocked with 5% (*w/v*) nonfat dry milk in Tris-buffered saline containing 0.1% (*v/v*) Tween-20 (TBS-T) at 4 °C overnight and incubated with primary antibodies at room temperature for 1.5 h. The membranes were washed three times with TBS-T and blotted with secondary antibodies conjugated with horseradish peroxidase at room temperature for 1.5 h, followed by washing three times in TBS-T. Immunoreactive proteins were visualized by an enhanced chemiluminescence procedure according to the protocol of the manufacturer (Santa Cruz Biotechnology, Santa Cruz, CA, USA) and exposed to X-ray films. Protein contents were normalized by reprobing the same membrane with anti-β-actin antibody. For β-actin detection, previously used membranes were soaked in stripping buffer (Gene Bio-Application Ltd., Hanagid, Israel) at room temperature for 20 min [25].

### 4.9. Statistical Analysis

Data are expressed as the mean ± S.D., unless otherwise specified. Statistical significance was assessed by two-tailed unpaired student’s *t*-test and *p* < 0.05 was considered statistically significant.

## 5. Conclusions

To the best of our knowledge, we reported, for the first time, that an extract of *P. pterocarpum* stem bark, SPP, effectively inhibited LDL oxidation. This plant suppressed the growth of human leukemia HL-60 and CCRF-SBA cancer cells, significantly inhibiting cell proliferation and increasing the activation of caspases 3 and 9. Treatment with SPP upregulated Bax protein expression and downregulated Bcl-2 protein expression in both HL-60 and CCRF-SBA cells. These results indicated that the stem bark of Vietnamese *P. pterocarpum* might represent a useful anti-oxidant of LDL and induce apoptosis in a human leukemia cancer cell line. Further experiments to identify active constituents and investigate the potential chemotherapeutic agents associated with leukemia cancer and atherosclerosis will be conducted.

## Figures and Tables

**Table 1:** Effects of LPP and SPP extracts on the formation of oxidation against Cu^2+^-mediated LDL oxidation.

Extracts/Compounds	Inhibition of LDL Oxidation IC_50_ (μg/mL) ^a^	
	Cu^2+^-Mediation	AAPH-Mediation
LPP	67.5 ± 3.6	>100
SPP	48.0 ± 5.2	40.5 ± 3.2
Quercetin ^b^	5.3 ± 0.8	10.5 ± 1.0
Caffeic acid ^b^	5.7 ± 1.4	40.7 ± 1.8
α-Tocopherol ^b^	25.8 ± 2.5	20.4 ± 1.0

^a^ IC_50_ values were determined by regression analysis and expressed as mean ± S.D. of three replicates. ^b^ Positive controls were expressed in μM.

**Table 2:** Cytotoxic activity of isolated compounds on human cancer cell lines.

Samples	Human cancer cells, IC_50_ μg/mL ^a^						
	MIA PACA2	PANC-1	A549	OVCAR8	KG	HL-60	CCRF-SBA
LPP	>500	>500	>300	>300	228.1 ± 6.8	146.0 ± 5.5	157.2 ± 10.4
SPP	167.5 ± 5.6	>300	244.1 ± 11.5	>300	255.0 ± 8.1	118.5 ± 3.8	120.7 ± 6.0
Camptothecin ^b^	3.2 ± 0.7	6.5 ± 0.8	2.05 ± 0.2	1.9 ± 0.1	0.8 ± 0.1	1.7 ± 0.4	11.7 ± 1.0

^a^ The results were obtained by triplicate experiments. ^b^ Positive anticancer reference (in μM).
